# Physicians’ opinions on barriers to patient counseling on physical activity in primary care: focus on physicians’ healthy exercise habits and knowledge about physical activity

**DOI:** 10.3389/fmed.2026.1711438

**Published:** 2026-03-11

**Authors:** Vilija Bite Fominiene, Martirija Fominaite, Saule Sipaviciene

**Affiliations:** 1 Department of Sports and Tourism Management, Lithuanian Sports University, Kaunas, Lithuania; 2 Faculty of Medicine, Lithuanian University of Health Sciences, Kaunas, Lithuania; 3 Department of Health Promotion and Rehabilitation, Lithuanian Sports University, Kaunas, Lithuania

**Keywords:** healthy exercise habits, knowledge, patient counseling, physical activity, physicians

## Abstract

**Introduction:**

Personal physical activity is recognized as an effective tool for the prevention and management of chronic diseases, where primary care professionals become important figures. The aim of this study was to reveal the opinions of physicians about the expression of barriers related to physical activity consultations for patients with chronic diseases, considering their own healthy exercise habits and the knowledge they have acquired during their studies and currently have about physical activity and its benefits in chronic diseases.

**Methods:**

A quantitative research strategy with cross-sectional design was used for study. The survey tool was designed to collect sociodemographic data, as well as data on primary care physicians’ self-reported barriers to providing physical activity (PA) – related recommendations to patients, their healthy exercise habits, and their knowledge of the effects of physical activity in managing chronic diseases.

**Results:**

The majority of the respondents (44.1%) have average healthy exercise habits, stated that they had received such knowledge only partially at university (98%–48.5%) or not at all (43%–21.3%) and now they have partially sufficient knowledge (109%–54.0%). The main obstacles preventing patients from providing recommendations about PA, according to the respondents, are lack of time at work (3.4 ± 0.817) and the patients themselves prefer pharmacological treatment (3.31 ± 0.790). Further analysis revealed that physicians’ healthy exercise habits and knowledge are significantly related to their opinion about the expression of barriers.

**Conclusion:**

Based on the results of the study, it can be concluded that the adequacy of physicians’ knowledge about the benefits of physical activity for patients with chronic diseases and their own healthy lifestyle habits have a significant impact on the expression of perceived barriers when counseling patients on PA issues. These data emphasize the need to improve medical curricula and professional development, strengthening physicians’ knowledge and skills in the field of PA, as well as promoting their own healthy lifestyle practices as an important factor for the successful integration of PA recommendations into primary health care.

## 1 Introduction

Adequate physical activity (PA) is associated with a reduced risk of premature death from major non-communicable chronic diseases (1–3). According to the most recent World Health Organization (4) guidelines on physical activity, individuals with chronic medical conditions should engage in moderate-intensity aerobic physical activity for at least 150–300 min per week or vigorous-intensity aerobic physical activity for at least 75–150 min per week, or an equivalent combination. They should also engage in muscle-strengthening exercises at least twice a week and multicomponent activities at least three times per week (4). However, despite the benefits of PA, the level of PA in society is insufficient. The prevalence of physical inactivity was 14.4% in 2008 and increased to 69.6% in 2015 (5). Based on an analysis of 1.9 million study participants, the global prevalence of physical inactivity was 27.5% in 2016 (6). According to WHO, more than a quarter of adults over the age of 18 worldwide are physically inactive (4). The 2020 WHO guidelines on physical activity and sedentary behavior established a contemporary, stepwise framework for prescribing physical activity (PA) in the general population. The central principle of the guidelines – “some physical activity is better than none” – emphasizes that measurable health benefits occur even below the recommended thresholds (4, 7). Consequently, physically inactive individuals are encouraged to begin with small, achievable amounts of activity and progressively increase duration, frequency, and intensity over time (7). This gradual progression model is essential for improving accessibility, safety, and long-term adherence, particularly among highly sedentary individuals and those with chronic conditions and supports adaptation based on functional capacity and baseline physical activity levels. By prioritizing early reductions in sedentarism and achievable increases in light activity, the WHO framework promotes engagement and sustainability, ultimately facilitating progression toward recommended PA levels (7). It is estimated that approximately 5 million deaths per year could be avoided if the world population were more physically active. Insufficient PA or physical inactivity can have many harmful consequences and lead to public health problems (8, 9). The trend toward inadequate PA is closely linked to increasing rates of coronary heart disease, type 2 diabetes, osteoporosis, colon cancer, and obesity (2, 10).

Because physicians are the primary care providers for patients’ health and wellbeing, this study focused on physicians working in primary care. A primary care physician (PCP) diagnoses diseases, prevents diseases, educates patients, and monitors outcomes for chronic diseases (9, 11). Primary care physicians could play a key role in promoting PA for their patients. PA counseling provided by primary care physicians could increase patients’ PA levels because physician advice is a powerful motivator for patients and influences patients’ healthy lifestyle behaviors (9, 12, 13). However, only a small proportion of physicians counsel their patients on physical activity. Evidence consistently shows that despite strong guideline recommendations, PA counseling remains underutilized in routine clinical care. National survey data from the United States and Canada indicate that only a minority of physicians regularly counsel patients about PA, confirming a persistent gap between recommendations and practice (14). A 2023 meta-analysis found that the overall prevalence of physical activity counseling in primary care was only 37.9% (15). The most common barrier to PA counseling cited by physicians is lack of time when dealing with multiple or complex medical problems (16). In addition to time pressure, clinicians frequently identify lack of reimbursement and insufficient institutional support as structural barriers that discourage PA counseling integration into standard care (17, 18). Studies show that many physicians feel inadequately prepared to prescribe PA or to tailor recommendations to specific patient conditions, and that familiarity with current PA guidelines is often limited (12, 16, 17, 19). Physicians also report perceived patient-related barriers, including concerns about low motivation, poor adherence, or limited patient interest in behavioral change (13).

In this context, important factors are related to PCPs’ attitudes, knowledge and behaviors, which are considered important in motivating patients to become more physically active, and there is a lack of research in this area. The aim of this study was to reveal the opinions of physicians about the expression of barriers related to physical activity consultations for patients with chronic diseases, considering their own healthy exercise habits and the knowledge they have acquired during their studies and currently have about physical activity and its benefits in chronic diseases.

## 2 Materials and methods

### 2.1 Participants and procedure

This study selected PCPs from Kaunas City Municipality hospitals and consultation clinics. The city has the largest number of practicing primary care physicians in Lithuania – 7.7 physicians per 10,000 patients, and has the largest university training such specialists. In this study, all primary care physicians working in these institutions were identified as the target population, i.e., a subset of the population of interest for a comprehensive study (20), which consisted of 424 PCPs. A convenience sampling technique was utilized for this study and sample size of 202 PCPs was calculated from this target population by using Raosoft ^®^ (Sample Size Calculator; Raosoft inc.) (21), with a confidence interval of 95%, 5% margins of error and 50% population proportion. Primary care physicians were invited to participate via publicly available institutional email addresses. A total of 358 eligible email addresses were identified in Kaunas city health care institutions. All identified physicians were invited to participate and those who responded voluntarily constituted the final study sample. The inclusion criteria were: (a) being a practicing primary care physician working with adult patients with non-communicable chronic diseases, including cardiovascular diseases, cancer, chronic respiratory diseases and diabetes, as defined by WHO, and b) providing routine care in primary care settings. The exclusion criterion was working exclusively with pediatric populations. Among the participants, there were 122 females (60.4%) and 80 males (39.6%).

For this study a quantitative research strategy with cross-sectional design was used. This study was approved by the Research Ethics Board of Lithuanian Sports University (protocol No. SMTEK-201, 2023.11.10). In order to ensure the subjects’ right to know and follow the principle of voluntariness, anonymity and confidentiality the subjects were informed before completing the questionnaire about the purpose of the study, procedures, potential risks and benefits, the approximate time needed to complete the survey (10–15 min) and the freedom of participants to discontinue participation at any time by giving informed consent on the first page of the questionnaire.

All data were collected via an online survey between October and December 2023. Participants were approached through personalized invitation using their work e-mail. The emails contained a direct link to an online survey.^1^ PCPs respondent individually from any device and Responses could be provided from any location and only one response per user was possible. All questions were set as mandatory, which prevented data missing.

### 2.2 Measures

The socio-demographic information of participants was collected through a self-designed questionnaire, which included gender, age, years of experience as PCP.

Physicians’ opinions about the expression of barriers that hinders them from providing recommendations related to physical activity (PA) to patients were assessed with seven items (*Patients are not interested in PA-promoting exercises; Patients prefer pharmacological treatment; There is a lack of validated recommendations/guidelines on PA; I lack time at work to additionally advise patients on PA; I personally lack specific knowledge about PA; The priority is to provide patients with recommendations that will help them abandon major harmful habits; Lack of evidence on the health benefits of physical activity*), taken from previously validated survey instruments (22–24). These items were rated using a four-point rating scale, includes the following: This is not a barrier, There is a small barrier, There is a medium barrier, There is a large barrier. The reliability analysis showed good internal consistency - Cronbach’s alpha for the subscale in this study was 0.82.

Subjects’ opinions about their healthy exercise habits were assessed using The “Health & Exercise” subscale of the Lifestyle and Habits Questionnaire-Brief (LHQ-B) (25). Here six items (e.g., “I participate in vigorous exercise like running, swimming, speed walking, or dance classes for at least 20–30 min a day and at least three times a week”) were rated on a five-point Likert scale ranging from 1 (never) to 5 (very often) and, healthy exercise habits groups (Poor, Average, and Good) were distinguished depending on the number of points scored. Internal consistency reliability of the LHQ-B “Health & Exercise” subscale was calculated using Cronbach’s alpha and McDonald’s omega. The subscale of the Lifestyle and Habits Questionnaire-Brief demonstrated good internal consistency (Cronbach’s α = 0.87; McDonald’s ω = 0.82) (26). The Exploratory Factor Analysis (EFA) was used to assess the construct structure of the LHQ-B subscale. The KMO value was 0.79 and Bartlett’s test of sphericity showed statistical significance [χ^2^ (15) = 468.19; *p* < 0.001], indicating that data is suitable for the factor analysis. The analysis identified a single-factor solution. This factor explained 45.7% of the total variance in the data. Most of the statements had good factor weights, and one statement had a lower weight, but was retained in order to maintain the original structure of the questionnaire.

The opinion of the subjects about their knowledge about the effects of physical activity on the organism of a person with chronic diseases was assessed with two questions. These questions, according to previous surveys (27, 28) were self-developed for this study. First of all, we were interested in whether the subjects received sufficient knowledge during their studies (*Did you receive sufficient knowledge about the effects of physical activity on the body in chronic diseases during your studies*?). Second, all subjects were asked to indicate the adequacy of their current knowledge related to PA (*Do you currently have sufficient knowledge to make physical activity recommendations for patients with chronic diseases?*). Subjects’ opinion about their knowledge of PA was rated with a three-point system (Did not get/Do not have, Partially got/have, Sufficiently got/have).

### 2.3 Statistic analysis

IBM SPSS version 29 (Statistical Package for the Social Sciences) for Windows was used for data analysis. Descriptive statistics – percentages, means, and standard deviations – were initially calculated to summarize the characteristics and responses of the study sample by key variables. Crosstabs were used to assess associations between categorical variables, and the chi-square (χ^2^) test was used to determine statistical significance. Z-scores, mean ranks, and rank sums were calculated to compare group differences when the data did not meet the assumptions of normality. The Mann–Whitney U test was used to compare two independent groups, and the Kruskal–Wallis *H*-test was used to compare more than two independent groups. In the presence of statistically significant results, *post hoc* analysis was performed to determine which groups differed in mean ranks. Statistical significance was set at *p* < 0.05.

## 3 Results

This study involved 202 participants (Table 1). Recruitment and inclusion of participants followed this sequence: primary care physicians working in Kaunas city health care institutions (*n* = 424) → physicians with publicly available institutional email addresses (*n* = 358) → physicians invited to participate (*n* = 358) → participants included in the final analysis (*n* = 202). All questionnaire items were mandatory therefore no items level missing data were present in the analyzed dataset. Among the respondents 122 (60.4%) were female, and 124 (61.4%) had more than 10 years’ work as PCP experience. After evaluating the participants’ *healthy exercise habits*, it was discovered that the majority of the respondents (44.1%) have average healthy exercise habits, and a similar number of participants reported either poor (26.7%) or good (29.2%) habits. Statistically significant differences were found in terms of gender, the results were presented in an older study (27). Meanwhile, when analyzing the work as PCP experience of physicians, no significant differences were found.

**TABLE 1:** Sample characteristics by gender and work experience (*N* = 202).

Characteristics	All *n* = 202	Gender			Work as PCP experience (years)		
		Male *n* = 80 (39.6%)	Female *n* = 122 (60.4%)	*P*-value	≤10 *n* = 78 (38.6%)	>10 *n* = 124 (61.4%)	*P*-value
**Healthy exercise habits *N* (%)**							
Good	59 (29.2)	21 (26.3)	38 (31.1)	<0.001	20 (25.6)	39 (31.5)	0.627
Average	89 (44.1)	26 (29.2)	63 (51.6)		35 (44.9)	54 (43.5)	
Poor	54 (26.7)	33 (41.3)	21 (17.2)		23 (29.5)	31 (25.0)	
**Evaluation of the adequacy of knowledge acquired during studies about the impact of physical activity on the body in cases of chronic illness, by group *N* (%)**							
Did not get knowledge	43 (21.3)	20 (25.0)	23 (18.9)	0.547	16 (20.5)	27 (21.8)	0.270
Partially got knowledge	98 (48.5)	38 (47.5)	60 (49.2)		43 (55.1)	55 (44.4)	
Sufficiently got knowledge	61 (30.2)	22 (27.5)	39 (32.0)		19 (24.4)	42 (33.9)	
**Assessment of the adequacy of currently available knowledge to provide physical activity recommendations for patients with chronic diseases, by group *N* (%)**							
Do not have knowledge	38 (18.8)	16 (20.0)	22 (18.0)	0.082	12 (15.4)	26 (21.0)	0.613
Partially have knowledge	109 (54.0)	36 (45.0)	73 (59.8)		44 (56.4)	65 (52.4)	
Sufficiently have knowledge	55 (27.2)	28 (35.0)	27 (22.1)		22 (28.2)	33 (26.6)	

After analyzing the knowledge gained during studies about the effects of PA on the body in chronic diseases, it was found that the majority of respondents stated that they had received such knowledge only partially (98%–48.5%) or not at all (43%–21.3%). Slightly less than a third of the respondents stated that they had received sufficient knowledge during their studies (61%–30.2%). No statistically significant differences were found in terms of gender and work experience. The adequacy of current knowledge enabling the provision of physical activity recommendations to patients with chronic diseases was assessed. It was found that the majority of respondents have either partially sufficient knowledge (109 respondents, 54.0%) or fully sufficient knowledge (55 respondents, 27.2%). Statistically significant differences were found in terms of gender (*p* = 0.082). A larger proportion of women compared to men believe that the knowledge that allows them to prescribe PA recommendations to patients with chronic diseases is partially sufficient for them, while men claim that such knowledge is completely sufficient for them. No statistically significant differences were found in terms of work experience.

After analyzing the strength of obstacles to providing PA recommendations to patients (Table 2), it was found that the greatest obstacle identified by the surveyed physicians was lack of time at work (3.40 ± 0.82) and patients’ own preference for pharmacological treatment (3.31 ± 0.79). Meanwhile, respondents do not consider the lack of evidence about the benefits of PA to be a major or moderate obstacle (1.91 ± 0.96). In the opinion of the respondents, the priority of providing patients with recommendations that will help them give up major harmful habits is also becoming a minor obstacle (2.26 ± 0.80).

**TABLE 2:** Expression of barriers to patients making recommendations related to physical activity (PA).

Barriers	Men	SD
B1 – patients are not interested in PA-promoting exercises	3.04	0.94
B2 – patients prefer pharmacological treatment	3.31	0.79
B3 – there is a lack of validated recommendations/guidelines on PA	2.56	0.90
B4 – lack of time at work	3.40	0.82
B5 – lack specific knowledge about PA	2.72	0.88
B6 – the priority is to provide patients with recommendations that will help them abandon major harmful habits	2.26	0.80
B7 – lack of evidence on the health benefits of physical activity	1.91	0.96

After analyzing the correlations between the expression of barriers and the gender and work experience of the subjects (Table 3), it was found that in barrier B2, statistically more men (*p* < 0.001) and those with less work experience (*p* = 0.003) consider this to be a barrier. B3 can also be considered obstacles to providing PA recommendations to patients (women are statistically more likely to be convinced of this (*p* = 0.034), and in B1 statistical differences were found between both gender (*p* = 0.007) and work experience (*p* = 0.022). This is more often identified as an obstacle by women and physicians with less work experience. B5 is statistically significantly identified as a barrier by women compared to men (*p* < 0.001). Meanwhile, the B6 is also considered a greater barrier by physicians with less work experience compared to physicians with more experience (*p* = 0.011).

**TABLE 3:** Relationships between the expression of barriers and the gender and work experience of the subjects.

Barrier	Gender					Work as PCP experience (years)					
	Male, *n* = 80	Female, *n* = 122		z	*P*	≤10, *n* = 78		>10, *n* = 124		z	*P*
Mean rank	Sum of ranks	Mean rank	Sum of ranks			Mean rank	Sum of ranks	Mean rank	Sum of ranks		
B188.86	7108.50	109.79	13394.50	–2.64	0.008	110.53	8621.00	95.82	11882.00	–1.84	0.065
B287.58	7006.50	110.63	13496.50	–3.00	0.003	118.64	9254.00	90.72	11249.00	–3.62	<0.001
B395.11	7609.00	105.69	12894.00	–1.33	0.184	106.51	8308.00	98.35	12195.00	–1.02	0.308
B493.14	7451.50	106.98	13051.50	–1.86	0.063	107.24	8365.00	97.89	12138.00	–1.25	0.210
B589.39	7151.00	109.44	13352.00	–2.53	0.011	98.19	7659.00	103.58	12844.00	–0.68	0.499
B6107.38	8590.00	97.65	11913.00	–1.24	0.215	116.59	9094.00	92.01	11409.00	–3.12	0.002
B7102.96	8237.00	100.54	12266.00	–0.31	0.759	106.49	8306.00	98.36	12197.00	–1.02	0.306

After analyzing the associations between barriers and knowledge gained during studies about the effects of PA on the body in chronic diseases (Table 4), associations were identified in all barriers except for the barrier B2 (*p* = 0.654). When comparing data on the expression of barriers according to groups of levels of knowledge received (did not receive such knowledge, received partially or received enough), statistically significant differences were identified. Respondents who received sufficient knowledge about the effects of PA on the body in chronic diseases during their studies, compared to respondents who received partial and insufficient knowledge, consider B3; B4; B5, and B6 to be a weaker obstacle to consulting patients on PA issues. Respondents who stated that during their studies they did not receive any knowledge about the effects of physical activity on the body in chronic diseases, compared to respondents who stated that they received such knowledge partially and received it sufficiently, consider B1 as a stronger obstacle preventing them from consulting patients on physical activity issues. Respondents who claim that they received knowledge partially during their studies, compared to those who claim that they received such knowledge sufficiently, consider B7 to be a stronger obstacle to counseling patients on PA issues.

**TABLE 4:** Comparison of barriers and knowledge gained during studies about the effects of physical activity (PA) on the body in chronic diseases, by groups.

Barriers	Groups of received knowledge levels	Mean rank	Kruskal Wallis Test			Comparable groups	Differences in averages		
			H	df	*P*		Mann-Whitney U	Z	*P*
B1	Did not get (g1)	124.58	13.17	2	0.001	g1–g2*	1624.000	–2.32	0.02
	Partially got (g2)	101.82				g2–g3	2474.500	–1.92	0.055
	Sufficiently got (g3)	84.71				g1–g3***	802.000	–3.56	<0.001
B2	Did not get (g1)	101.40	0.85	2	0.654	No differences			
	Partially got (g2)	104.60							
	Sufficiently got (g3)	96.59							
B3	Did not get (g1)	114.95	7.55	2	0.023	g1–g2	1891.500	–1.02	0.308
	Partially got (g2)	105.07				g2–g3*	2423.500	–2.12	0.034
	Sufficiently got (g3)	86.28				g1–g3*	948.500	–2.52	0.012
B4	Did not get (g1)	115.86	10.97	2	0.004	g1–g2	1898.000	–1.11	0.267
	Partially got (g2)	106.02				g2–g3*	2337.000	–2.56	0.01
	Sufficiently got (g3)	84.11				g1–g3**	903.000	–2.99	0.003
B5	Did not get (g1)	112.49	15.18	2	<0.001	g1–g2	2073.500	–0.16	0.873
	Partially got (g2)	110.97				g2–g3***	2027.50	–3.61	<0.001
	Sufficiently got (g3)	78.54				g1–g3**	872.50	–3.08	0.002
B6	Did not get (g1)	118.43	15.02	2	<0.001	g1–g2	1879.00	–1.10	0.273
	Partially got (g2)	107.57				g2–g3**	2166.00	–3.13	0.002
	Sufficiently got (g3)	79.81				g1–g3***	811.50	–3.55	<0.001
B7	Did not get (g1)	102.13	12.50	2	0.002	g1–g2	1875.00	–1.09	0.275
	Partially got (g2)	113.48				g2–g3***	2047.00	–3.55	<0.001
	Sufficiently got (g3)	81.81				g1–g3	1052.50	–1.90	0.058

*p < 0.05;

**p < 0.005;

***p < 0.001.

After analyzing the correlations between the indicated barriers and the currently available knowledge that allows prescribing PA recommendations to patients with chronic diseases (Table 5), correlations were found in all groups, except for the comparison in barrier B2 (*p* = 0.496). This means that respondents in all groups see this as a barrier equally strongly (see Table 2). Respondents who state that they have insufficient knowledge to provide PA recommendations to patients with chronic diseases, more often than those who have partially sufficient and completely sufficient such knowledge, indicate as a stronger obstacle to consulting patients on PA issues are B4, B5, B6. Respondents who state that they have completely enough knowledge identify B3 and B7 as a weaker obstacle, compared to those who claim to have partially sufficient and insufficient knowledge. Respondents who state that they have completely enough knowledge identify B1 as a stronger obstacle, compared to those who claim to have partially sufficient knowledge.

**TABLE 5:** Comparison of barriers and available knowledge to prescribe physical activity (PA) recommendations to patients with chronic diseases across groups.

Barriers	Groups of sufficiency of available knowledge	Mean rank	Kruskal Wallis Test			Comparable groups	Differences in averages		
			H	df	*P*		Mann-Whitney U	Z	*P*
B1	Do not have (g1)	104.04	7.413	2	0.025	g1–g2	1849.00	–1.03	0.301
	Partially have (g2)	92.63				g2–g3	2253.00	–2.75	0.006
	Sufficiently have (g3)	117.32				g1–g3	919.50	–1.06	0.289
B2	Do not have (g1)	110.55	1.401	2	0.496	No differences			
	Partially have (g2)	98.72							
	Sufficiently have (g3)	100.76							
B3	Do not have (g1)	114.58	9.085	2	0.011	g1–g2	1897.00	–0.82	0.412
	Partially have (g2)	106.29			g2–g3		2301.50	–2.56	0.011
	Sufficiently have (g3)	82.97			g1–g3		722.00	–2.64	0.008
B4	Do not have (g1)	124.82	9.713	2	0.008	g1–g2	1496.00	–2.92	0.004
	Partially have (g2)	97.35				g2–g3	2875.00	–0.47	0.638
	Sufficiently have (g3)	93.62				g1–g3	734.00	–2.86	0.004
B5	Do not have (g1)	145.58	32.038	2	<0.001	g1–g2	1000.00	–5.08	<0.001
	Partially have (g2)	95.74				g2–g3	2554.00	–1.65	0.099
	Sufficiently have (g3)	82.45				g1–g3	441.00	–4.93	<0.001
B6	Do not have (g1)	123.63	7.742	2	0.021	g1–g2	1515.50	–2.65	0.008
	Partially have (g2)	96.89				g2–g3	2945.00	–0.20	0.845
	Sufficiently have (g3)	95.35				g1–g3	759.50	–2.38	0.017
B7	Do not have (g1)	119.57	13.704	2	0.001	g1–g2	1805.00	–1.24	0.216
	Partially have (g2)	106.28				g2–g3	2211.00	–2.95	0.003
	Sufficiently have (g3)	79.55				g1–g3	624.50	–3.55	<0.001

After analyzing the associations between barriers and physicians’ healthy exercise habits (Table 6), associations were identified in all barriers except B6 (*p* = 0.740). Analyzing the connections within the groups revealed that respondents demonstrating good healthy exercise habits, compared to those demonstrating poor and average healthy exercise habits, consider B1 to be a stronger obstacle to counseling patients on PA issues - good PA skills determine their attitude that patients are not interested in PA. Respondents demonstrating poor healthy exercise habits, compared to those demonstrating good and average healthy exercise habits, consider B3 and B4 as weaker barriers to counseling patients on PA, i.e., those with better healthy exercise skills may have a better understanding of what is needed in terms of recommendations and guidelines related to PA. Respondents demonstrating average healthy exercise habits, compared to those demonstrating poor and good healthy exercise habits, consider B7 and B5 to be stronger barriers to counseling patients on PA issues. Respondents demonstrating average healthy exercise habits, compared to those demonstrating poor healthy exercise habits, also consider B2 to be a stronger obstacle to counseling patients on PA issues.

**TABLE 6:** Comparison of barriers and primary care physicians (PCPs) healthy exercise habits, by groups.

Barriers	Healthy exercise habits groups	Mean rank	Kruskal Wallis Test			Comparable groups	Differences in averages		
			H	df	*P*		Mann-Whitney U	Z	*P*
B1	Poor (g1)	85.67	10.16	2	0.006	g1–g2	2065.00	–1.49	0.136
	Average (g2)	99.81				g2–g3	2137.00	–2.04	0.041
	Good (g3)	118.54				g1–g3	1076.00	–3.15	0.002
B2	Poor (g1)	84.41	8.07	2	0.018	g1–g2	1778.00	–2.85	0.004
	Average (g2)	110.28				g2–g3	2469.00	–0.69	0.493
	Good (g3)	103.90				g1–g3	1295.00	–1.84	0.065
B3	Poor (g1)	84.64	8.07	2	0.018	g1–g2	1946.00	–2.03	0.042
	Average (g2)	103.53				g2–g3	2349.00	–1.15	0.252
	Good (g3)	113.87				g1–g3	1139.50	–2.73	0.006
B4	Poor (g1)	79.87	12.95	2	0.002	g1–g2	1675.50	–3.37	<0.001
	Average (g2)	110.13				g2–g3	2584.50	–0.19	0.848
	Good (g3)	108.27				g1–g3	1152.50	–2.78	0.005
B5	Poor (g1)	92.44	6.65	2	0.036	g1–g2	1904.00	–2.23	0.026
	Average (g2)	112.78				g2–g3	2121.00	–2.08	0.038
	Good (g3)	92.78				g1–g3	1583.00	–0.06	0.951
B6	Poor (g1)	103.05	0.60	2	0.740	No differences			
	Average (g2)	98.25							
	Good (g3)	104.99							
B7	Poor (g1)	85.64	14.60	2	<0.001	g1–g2	1605.00	–3.52	<0.001
	Average (g2)	117.98				g2–g3	1956.50	–2.76	0.006
	Good (g3)	91.15				g1–g3	1534.50	–0.38	0.706

## 4 Discussion

This study identified three key findings, which reveal that primary care physicians perceived barriers to PA counseling are significantly associated with both their self-reported knowledge and PA and their personal healthy exercise habits. It was also revealed that lack of time and patients’ preference for pharmacological treatment represent the most prominent barriers across all PCP groups, and physicians with higher levels of PA knowledge and healthier exercise habits tend to perceive fewer knowledge and system related barriers to PA counseling. Understanding PCPs views is critical because physicians are often the first point of contact for patients and play a pivotal role in promoting healthy lifestyle behaviors, including regular physical activity, which is essential for the prevention and management of chronic conditions such as diabetes, cardiovascular disease, and obesity (7, 13, 18).

We tried to find a connection between the barrier “lack of time” and physicians’ knowledge about PA level and the physicians own healthy exercise habits. The results of our study showed that physicians indicate lack of time at work as a weaker obstacle preventing them from consulting patients on PA issues, having received sufficient knowledge about PA effects on the body in chronic diseases during their studies, compared to respondents who partially and insufficiently received knowledge. Meanwhile, currently insufficient knowledge of physicians is associated with lack of time at work as a stronger obstacle preventing them from consulting patients on PA issues, compared to respondents who partially and sufficiently have knowledge. We found that physicians who demonstrate their weak healthy exercise habits, indicate lack of time at work as a weaker obstacle preventing them from consulting patients on PA issues, compared to respondents with average or excellent healthy exercise habits.

### 4.1 The expression of barriers to PCP provided PA consultations for patients compared to physicians’ knowledge

Our study results showed that only one third (30.2%) of physicians received sufficient knowledge about the effects of PA on the body in chronic diseases during their studies, with the majority of respondents stating that they received such knowledge only partially (48.5%) or did not receive it at all (21.3%). Gender and work experience did not influence the results. Insufficient knowledge about PA recommendations during studies has been indicated as a significant obstacle to PA counseling practice in previous studies (8, 17). A recent study indicated that only 21.8% of PCPs were trained about PA counseling during medical school or residency programs (29). Therefore, one of the main obstacles reported by 38.1% of physicians that prevented them from providing PA counseling to their patients was the lack of adequate training about PA (29). Other researchers reported that 84.8% of physicians cited a lack of exercise education in medical school (25). Pugh et al. (30) found that medical students are aware of the benefits of PA in treating diseases, but lack the confidence to provide advice to patients due to their own lack of knowledge of current PA guidelines.

Having analyzed the correlations between barriers to physical activity consultations for patients and knowledge obtained during studies about the effects of PA on the body in chronic diseases, we found significant differences according to the groups of knowledge levels obtained (did not receive such knowledge, received partially or received enough). Physicians who received sufficient knowledge about the effects of PA on the body in chronic diseases during their studies indicated as weaker barriers preventing them from consulting patients on PA issues: “lack of approved recommendations/guidelines on physical activity,” “lack of time at work,” “lack of specific knowledge about physical activity,” “priority of providing patients with recommendations that will help them give up major harmful habits.” Physicians who stated that they did not receive knowledge about the effects of PA on the body in chronic diseases during their studies considered “patients are not interested in exercises that promote physical activity” as stronger barriers preventing them from consulting patients on PA issues. Physicians who claim to have partially acquired knowledge during their studies consider “the lack of approved recommendations/guidelines on physical activity” to be a stronger obstacle to counseling patients on PA issues.

Our study results showed that the current knowledge of physicians that allows them to prescribe physical activity recommendations to patients with chronic diseases was indicated by the following respondents: 18.8% of the knowledge is insufficient, 27.2% of the knowledge is completely sufficient and 54% of the knowledge is partially sufficient. A larger proportion of female PCPs, compared to men, believe that their knowledge that allows them to prescribe PA recommendations to patients with chronic diseases is partially sufficient, while men say that such knowledge is completely sufficient. No significant differences were found in terms of work experience. According to another recent study, 60.94% of PCPs’ admitted that their knowledge about PA recommendations to patients is from “low” to “moderate” level (9). The relatively low level of knowledge and accuracy in describing PA guidelines in a previous study was surprising, as only 13% of general practitioners correctly described PA recommendations (including frequency, duration, and intensity), although most respondents believed they had sufficient knowledge (10). The results of a recent study showed that only 5.4% of physicians had excellent knowledge, 59.2% had poor knowledge, and 33.3% had good knowledge of PA recommendations (29). In another study it was indicated that physicians were confident in their ability to provide physical consultations, but it was found that 59.2% of physicians had a poor level of knowledge of recommended PA guidelines (16). An even higher proportion of 78.3% of physicians surveyed in another study indicated a lack of personal knowledge (25). In a recent study, 25% of physicians reported that they had some education in the field of PA counseling, which made them more confident and able to advise patients (9). In one study, only 8.3% of physicians self-rated their knowledge of how to perform PA consultations as excellent, and a significant association was found between the level of knowledge in providing PA consultations (31).

After analyzing the correlations between barriers to physical activity counseling for patients and the current knowledge about the effects of PA on the body in chronic diseases, we found significant differences according to the groups of knowledge levels (did not receive such knowledge, received partially or received enough). Physicians who stated that there was insufficient knowledge to provide PA recommendations to patients with chronic diseases, indicated stronger barriers preventing them from counseling patients on PA issues such as: “lack of time at work,” “lack of specific knowledge about physical activity,” “priority to provide patients with recommendations that will help them give up major harmful habits.” Physicians who stated that they had sufficient knowledge indicated stronger barriers such as: “entsatipare not interested in PA-promoting exercises” and considered weaker barriers such as: “lack of evidence about the health benefits of physical activity” and “lack of approved recommendations/guidelines about physical activity.”

These results have practical implications for medical education and professional development. Structured training in undergraduate and postgraduate curricula, complemented by continuing medical education (CME) and guideline-based workshops, can enhance physicians’ knowledge, confidence, and counseling behavior (13, 18, 32, 33). System-level supports, such as clinical decision tools, electronic health record prompts, and referral pathways, may further facilitate the integration of PA counseling into routine practice.

For future research, longitudinal studies should evaluate whether educational interventions and system-level strategies lead to sustained increases in PA counseling and measurable improvements in patient outcomes. Additionally, implementation studies could clarify how best to embed PA counseling training into curricula and professional development frameworks to overcome persistent barriers.

Primary care physicians struggle with their own perceptions and beliefs that they lack sufficient knowledge about PA counseling recommendations or that counseling is ineffective in changing patients’ health-promoting behaviors (10, 12, 16, 34). Lack of knowledge is a significant barrier to PA counseling. PCPs often lack the knowledge and skills to provide effective physical activity counseling (35, 36). Our findings and those of others suggest that medical school curricula need to be modified to include training in lifestyle change and PA promotion. PCPs who have previously been educated in providing PA recommendations are more capable and confident in providing PA (including exercise frequency, intensity, duration, and type) counseling to patients (9). Researchers indicate that physicians with good or excellent knowledge of PA guidelines and recommendations are more likely to promote PA for children, while those with excellent knowledge are more likely to value PA for pregnant women (30).

### 4.2 The expression of barriers to patients’ PA consultations provided by PCPs compared to the PA level of PCPs

Our previous study showed that the majority of the respondents 44.1% had average, 26.7% had weak and 29.2% had excellent healthy exercise habits (31). In a recent study conducted by other researchers, it was revealed that a significant proportion of physicians are physically inactive: 25.3% inactive, 24.1% moderately inactive, 25.3% moderately active, 25.3% active (37). Studies show a positive relationship between physicians’ demonstrated PA habits and PA counseling to patients (38). Physicians who are inactive themselves are less likely to provide advice on physical activity than their active colleagues, so physically active physicians may be ideal role models in communicating the importance of PA and advice to their patients (9, 16). Physically active physicians may be more likely to provide advice to their patients based on their personal experiences (9).

We found significant differences in the association between barriers to physical activity counseling for patients and physicians’ healthy exercise habits. Physicians who demonstrate excellent healthy exercise habits consider “patients are not interested in physical activity-promoting exercises” as a stronger barrier to counseling patients on PA issues, i.e., excellent physicians’ PA skills determine their perception that patients are not interested in PA. Physicians who demonstrate weak healthy exercise habits consider “lack of validated recommendations/guidelines on physical activity” and “lack of time at work” as weaker barriers to counseling patients on PA issues. Thus, physicians with better healthy exercise skills may better understand what the patient needs in terms of recommendations and guidelines related to PA. Physicians demonstrating average healthy exercise habits consider “lack of validated recommendations/guidelines on physical activity,” “lack of specific knowledge about physical activity” and “patients prefer pharmacological treatment” as stronger barriers to counseling patients on PA issues.

After analyzing the relationship between the expression of barriers and the work experience of physicians, we found that the barriers such as “patients themselves prefer pharmacological treatment,” “patients’ lack of interest in PA-promoting exercises,” “priority in providing patients with recommendations that will help them give up major harmful habits,” were also considered greater obstacles by physicians with less work experience (<10 years) compared to physicians with more experience (>10 years). Other researchers indicate that 3–5 years of work experience compared to 11 and <20 years of experience had a significant impact on physician’s recommendations for patients’ lifestyle regarding PA (39).

After analyzing the correlations between the expression of barriers and the gender of the subjects, it was found that the obstacle “patients themselves prefer pharmacological treatment” is statistically more frequently mentioned by male physicians. “Lack of specific knowledge about physical activity,” “lack of approved recommendations/guidelines about physical activity” and “patients’ lack of interest in PA-promoting exercises” are statistically significantly more often mentioned as obstacles by women compared to men. Other researchers have also identified significant barriers to PA counseling practice: the lack of “standard PA guidelines for counseling patients” and “patients prefer pharmaceutical interventions” (8, 26). According to Fowles et al., the most influential barrier (97.8%) was “patients not interested in exercise,” as well as a significant barrier (95.7%) was “lack of guidance in exercise for those with chronic disease” and 91.3% “other lifestyle changes more important.” Slightly smaller barriers were indicated by 78.3% “patient prefer medication management” and 71.7% “lack of evidence for effectiveness of exercise” (25).

Our study results showed that PCPs face a variety of challenges in providing physical activity counseling. One of the main barriers to providing physical activity counseling in primary care was the lack of sufficient time to effectively counsel patients. This major barrier has been identified by other researchers (12, 16, 25, 31, 34, 40, 41). Other researchers report varying results from studies indicating that 50%, 60.9%, 61%, 70.1%, or 95.7% of physicians believe that insufficient time hinders consultation with patients about PA and is a significant barrier (10, 25, 30, 34, 40). Patient visits are usually quite short and consultations often cover a wide range of patient health issues (35).

We tried to find a connection between the barrier “lack of time” and physicians knowledge about PA level and the physicians own healthy exercise habits. The results of our study showed that physicians indicate lack of time at work as a weaker obstacle preventing them from consulting patients on PA issues, having received sufficient knowledge about PA effects on the body in chronic diseases during their studies, compared to respondents who partially and insufficiently received knowledge. Meanwhile, currently insufficient knowledge of physicians is associated with lack of time at work as a stronger obstacle preventing them from consulting patients on PA issues, compared to respondents who partially and sufficiently have knowledge. We found that physicians who demonstrate their weak healthy exercise habits, indicate lack of time at work as a weaker obstacle preventing them from consulting patients on PA issues, compared to respondents with average or excellent healthy exercise habits.

According to the results of this study, physicians who are sufficiently physically active and have an adequate level of education in physical activity for chronic diseases are more motivated to provide recommendations for PA to their patients. Physicians also believe in their ability to provide more reliable and effective advice if they were sufficiently physically active (30). Due to different methods of PA advice, practical guidelines for PA advice in primary care should be developed (15) Consultation on PA by primary care physicians is one of the strategies to promote health-promoting lifestyles and positive patient behavior changes in the larger population (9).

#### 4.2.1 Strengths of the study

One of the strengths of this study is that it provides an empirical evidence from Eastern Europe, regions that are still underrepresented in the scientific literature on physical activity counseling in primary care settings. This helps to fill a gap in literature by clearly linking primary care physicians’ knowledge and professional experience with their perceived barriers to physical activity counseling. Such an integrated approach provides practical insights that are directly relevant to medical education and health care policies aimed at promoting physical activity among patients with chronic diseases.

#### 4.2.2 Limitation of the study

The use of convenience sample from a single urban area limits the generalizability of the findings to other regions, rural areas. Also, in this study, both physicians’ knowledge of physical activity and their own lifestyle habits were assessed using self-report measures rather than objective assessments. As a result, the healthy exercise habits reported by the subjects themselves may not reflect the real situation related to PCPs activity, and perceived knowledge may not accurately reflect the actual practice of providing evidence-based physical activity counseling. Also, the study did not assess the actual counseling practices of physicians or patient-level outcomes, nor did it examine system-level factors such as institutional policies, reimbursement mechanisms, or referral pathways. These unmeasured factors may play an important role in shaping physical activity counseling practices and should be addressed in future research.

## 5 Conclusion

This study shows that primary care physicians’ knowledge of physical activity and their own lifestyle habits influence how they perceive barriers to counseling patients with chronic diseases. Physicians with sufficient knowledge reported fewer barriers related to guidelines, time, and experience, while those with less knowledge perceived these barriers more strongly. Personal exercise habits were also associated with attitudes toward counseling. Physically active PCPs were more likely to highlight patients’ lack of motivation as a barrier, while less active PCPs were less likely to highlight lack of time or PA guidelines as a barrier. Female physicians and those with less professional experience were more likely to report knowledge-related barriers. In all groups, the most important barriers were lack of consultation time and patients’ preference for pharmacological treatment. These results highlight the need to reflect on the content of medical curricula and areas of continuing professional development, including outcomes related to the benefits of physical activity. It would also be worthwhile to promote healthy lifestyle practices among physicians in order to support the integration of physical activity counseling into primary health care.

## Data Availability

The raw data supporting the conclusions of this article will be made available by the authors, without undue reservation.

## Footnotes

1
www.apklausa.lt

## References

[1] Lee I Shiroma E Lobelo F Puska P Blair S Katzmarzyk P. Effect of physical inactivity on major non-communicable diseases worldwide: an analysis of burden of disease and life expectancy. *Lancet.* (2012) 380: 219–29. 10.1016/S0140-6736(12)61031-9 22818936 PMC3645500

[2] Kyu H Bachman V Alexander L Mumford J Afshin A Estep K et al. Physical activity and risk of breast cancer, colon cancer, diabetes, ischemic heart disease, and ischemic stroke events: systematic review and dose-response meta-analysis for the Global Burden of Disease Study 2013. *BMJ.* (2016) 354:i3857. 10.1136/bmj.i3857 27510511 PMC4979358

[3] Ekelund U Tarp J Steene-Johannessen J Hansen B Jefferis B Fagerland M et al. Dose-response associations between accelerometry measured physical activity and sedentary time and all cause mortality: systematic review and harmonised meta-analysis. *BMJ.* (2019) 366:l4570. 10.1136/bmj.l4570 31434697 PMC6699591

[4] World Health Organization. *WHO Guidelines on Physical Activity and Sedentary Behaviour*. Geneva: World Health Organization. (2020) 33369898

[5] Gonzalez-Viana A Violan Fors M Castell Abat C Castell Abat C Rubinat Masot M Oliveras L et al. Promoting physical activity through primary health care: the case of Catalonia. *BMC Public Health.* (2018) 18:968. 10.1186/s12889-018-5773-2 30075720 PMC6090750

[6] Guthold R Stevens G Riley L Bull F. Worldwide trends in insufficient physical activity from 2001 to 2016: a pooled analysis of 358 population-based surveys with 1.9 million participants. *Lancet Glob Health.* (2018) 358:1077–1086. 10.1016/S2214-109X(18)30357-7 30193830

[7] Bull F Al-Ansari S Biddle S Borodulin K Buman M Cardon G et al. World health organization 2020 guidelines on physical activity and sedentary behaviour. *Br J Sports Med.* (2020) 54:1451–62. 10.1136/bjsports-2020-102955 33239350 PMC7719906

[8] Saad H Low P Jamaluddin R Chee H. Level of physical activity and its associated factors among primary healthcare Workers in Perak, Malaysia. *Int J Environ Res Publ Health.* (2020) 17:5947. 10.3390/IJERPH17165947 32824361 PMC7459827

[9] Lee A Muhamad R Tan V. Physically active primary care physicians consult more on physical activity and exercise for patients: a public teaching-hospital study. *Sports Med Health Sci.* (2023) 6:82–8. 10.1016/j.smhs.2023.11.002 38463668 PMC10918360

[10] Douglas F Torrance N van Teijlingen E Meloni S Kerr A. Primary care staff’s views and experiences related to routinely advising patients about physical activity. A questionnaire survey. *BMC Public Health.* (2006) 6:138. 10.1186/1471-2458-6-138. 16719900 PMC1523207

[11] Katon W von Korff M Lin E Simon G. Rethinking practitioner roles in chronic illness: the specialist, primary care physician, and the practice nurse. *Gen Hosp Psychiatr.* (2001) 23:138–44. 10.1016/S0163-8343(01)00136-0 11427246

[12] Elley C Kerse N Arroll B Robinson E. Effectiveness of counselling patients on physical activity in general practice: cluster randomised controlled trial. *BMJ.* (2003) 32:793–6. 10.1136/bmj.326.7393.793 12689976 PMC153098

[13] Orrow G Kinmonth A Sanderson S Sutton S. Effectiveness of physical activity promotion based in primary care: systematic review and meta-analysis of randomised controlled trials. *BMJ.* (2012) 344:e1389. 10.1136/bmj.e1389 22451477 PMC3312793

[14] Shuval K Leonard T Drope J Katz D Patel A Melissa Maitin-Shepard M et al. Physical activity counseling in primary care: Insights from public health and behavioral economics. *CA Cancer J Clin.* (2017) 67:233–44. 10.3322/caac.21394 28198998

[15] Wattanapisit A Lapmanee S Chaovalit S Lektip C Chotsiri P. Prevalence of physical activity counseling in primary care: a systematic review and meta-analysis. *Health Promot Perspect.* (2023) 13:254–66. 10.34172/hpp.2023.31 38235006 PMC10790122

[16] Smith A Borowski L Liu B Galuska D Signore C Klabunde C. et al. U.S. primary care physicians’ diet-, physical activity-, and weight-related care of adult patients. *Am J Prev Med.* (2011) 41:33–42. 10.1016/j.amepre.2011.03.017 21665061 PMC3142674

[17] Hébert E Caughy M Shuval K. Primary care providers’ perceptions of physical activity counselling in a clinical setting: a systematic review. *Br J Sports Med.* (2012) 46:625–31. 10.1136/bjsports-2011-090734 22711796

[18] O’Brien M Shields C Oh P Fowles J. Health care provider confidence and exercise prescription practices of Exercise is medicine Canada workshop attendees. *Appl Physiol Nutr Metab.* (2017) 42:384–90. 10.1139/apnm-2016-0413 28177736

[19] Cardinal B Park E Kim M Cardinal M. If exercise is medicine, where is exercise in medicine? Review of US medical education curricula. *J Phys Act Health.* (2015) 12:1336–43. 10.1123/jpah.2014-0316. 25459966

[20] Willie M. Differentiating between population and target population in research studies. *Int. J. Med. Sci. Clin. Res. Stud.* (2022) 2;521–3. 10.47191/ijmscrs/v2-i6-14

[21] Raosoft. *Sample Size Calculator by Raosoft Inc.* (2023). Available online at: http://www.raosoft.com/samplesize.html (Accessed August 22, 2023)

[22] Al-Baker W Al-Dashshan A Chehab M Selim N Eljack I. Profile of physical activity, perceived barriers and related counselling practices of primary health care physicians in Qatar: cross-sectional study. *J Community Med Public Health.* (2020) 4:176. 10.29011/2577-2228.100076

[23] Beni Yonis O Saadeh R Chamseddin Z Alananzeh H. Exercise counseling by primary care physicians in jordan-A preliminary study. *J Prim Care Community Health.* (2020) 11:2150132720946947. 10.1177/2150132720946947 32713234 PMC7385832

[24] Fowles J O’Brien M Solmundson K Oh P Shields C. Exercise is medicine Canada physical activity counselling and exercise prescription training improves counselling, prescription, and referral practices among physicians across Canada. *Appl Physiol Nutr Metab.* (2018) 43:535–9. 10.1139/apnm-2017-0763 29316409

[25] Dinzeo T Thayasivam U Sledjeski E. The development of the lifestyle and habits questionnaire-brief version: relationship to quality of life and stress in college students. *Prev Sci.* (2014) 15:103–14. 10.1007/s11121-013-0370-1 23417669

[26] Yockey RD. *SPSS Demystified: A Simple Guide and Reference* (3rd ed.). Milton Park: Routledge (2017). 10.4324/9781315268545

[27] Burdick L Mielke G Parra D Gomes G Florindo A Bracco M et al. Physicians’, nurses’ and community health workers’ knowledge about physical activity in Brazil: a cross-sectional study. *Prev Med Rep.* (2015) 2:467–72. 10.1016/j.pmedr.2015.06.001 26844104 PMC4721435

[28] O’Brien S Prihodova L Heffron M Wright P. Physical activity counselling in Ireland: a survey of doctors’ knowledge, attitudes and self-reported practice. *BMJ Open Sport Exerc Med.* (2019) 5:e000572. 10.1136/bmjsem-2019-000572 31423324 PMC6677951

[29] Alahmed Z Lobelo F. Correlates of physical activity counseling provided by physicians: a cross-sectional study in Eastern Province, Saudi Arabia. *PLoS One.* (2019) 14:e0220396. 10.1371/journal.pone.0220396 31344113 PMC6657910

[30] Pugh G O’Halloran P Blakey L Leaver H Angioi M. Integrating physical activity promotion into UK medical school curricula: testing the feasibility of an educational tool developed by the Faculty of sports and exercise medicine. *BMJ Open Sport Exerc Med.* (2020) 6:e000679. 10.1136/bmjsem-2019-000679 32547778 PMC7279672

[31] Fominienë V Fominaitë M Sipavièienë S. Physical activity knowledge and personal habits with recommendations for patients: self-assessment by primary care physicians. *Healthcare.* (2024) 12:1131. 10.3390/healthcare12111131 38891205 PMC11172234

[32] Shivgulam M Petterson J Pellerine L Kivell M Wilson T Theou O et al. Effectiveness of physical activity counselling and exercise prescription education among medical students: a systematic review. *Can Med Educ J.* (2024) 15:95–112. 10.36834/cmej.77065. 39588016 PMC11586027

[33] Omura J Bellissimo M Watson K Loustalot F Fulton J Carlson S. Primary care providers’ physical activity counseling and referral practices and barriers for cardiovascular disease prevention. *Prev Med.* (2018) 108:115–22. 10.1016/j.ypmed.2017.12.030 29288783 PMC5870116

[34] Lewis K Gudzune K. Overcoming challenges to obesity counseling: suggestions for the primary care provider. *J Clin Outcomes Manag.* (2014) 21:123–33.

[35] Tulloch H Fortier M Hogg W. Physical activity counseling in primary care: who has and who should be counseling?. *Patient Educ Couns.* (2006) 64:6–20. 10.1016/j.pec.2005.10.010 16472959

[36] Alolayan A Alsubhi S. Physical activity assessment of physicians in primary healthcare centers in Makkah, Saudi Arabia. *Cureus.* (2024) 16:e67659. 10.7759/cureus.67659 39314599 PMC11418318

[37] Lobelo F de Quevedo I. The evidence in support of physicians and health care providers as physical activity role models. *Am J Lifestyle Med.* (2014) 10:36–52. 10.1177/155982761352012 26213523 PMC4511730

[38] Lobelo F Stoutenberg M Hutber A. The exercise is medicine global health initiative: a 2014 update. *Br J Sports Med.* (2014) 48:1627–33. 10.1136/bjsports-2013-093080 24759911

[39] Omura J Watson K Loustalot F Fulton J Carlson S. Primary care providers’ awareness of physical activity-related intensive behavioral counseling services for cardiovascular disease prevention. *Am J Health Promot.* (2019) 33:208–16. 10.1177/0890117118784226 29962209 PMC9770807

[40] Abramson S Stein J Schaufele M Frates E Rogan S. Personal exercise habits and counseling practices of primary care physicians: a national survey. *Clin J Sport Med.* (2000) 10:40–8. 10.1097/00042752-200001000-00008 10695849

[41] AuYoung M Linke S Pagoto S Buman M Craft L et al. Integrating physical activity in primary care practice. *Am J Med.* (2016) 129:1022–9. 10.1016/j.amjmed.2016.02.008 26953063

